# Supplementary figures and images for: The βI domain promotes active β1 integrin clustering into mature adhesion sites
Source: Life Sci Alliance. 2022 Nov 21;6(2):e202201388. doi: 10.26508/lsa.202201388 (PMC9679427; doi:10.26508/lsa.202201388)

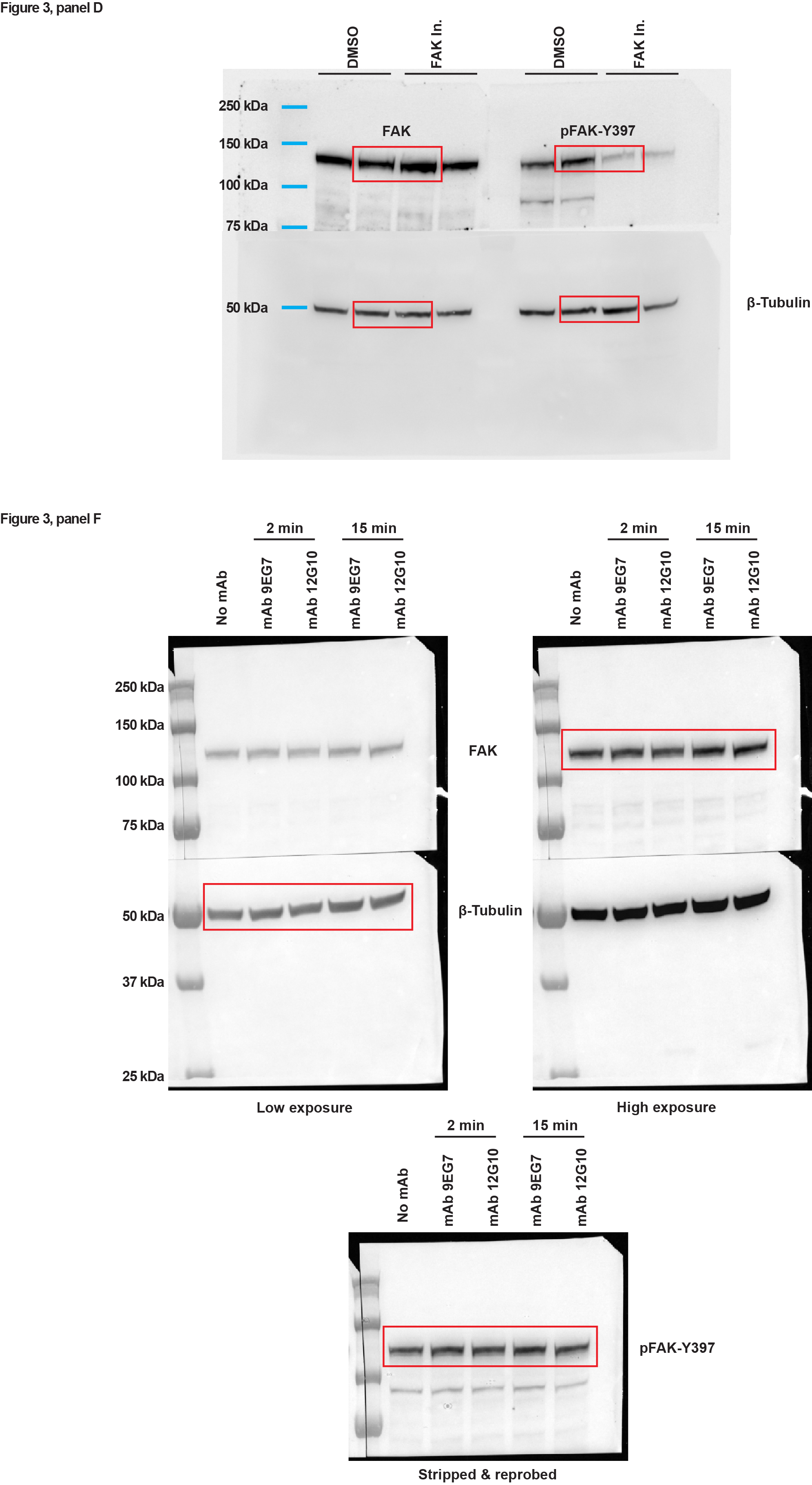

Supplement: Supplementary file 7 [file LSA-2022-01388_SdataF3.2.jpg]
